# Bomb blast: imaging findings, treatment and clinical course of extremity traumas

**DOI:** 10.1186/s12873-021-00421-7

**Published:** 2021-03-06

**Authors:** Mehmet Tahtabasi, Sadettin Er, Recep Karasu, Erhan Renan Ucaroglu

**Affiliations:** 1 Department of Radiology, University of Health Sciences-Somalia Turkey Recep Tayyip Erdogan Education and Research Hospital, Mogadishu, Somalia; 2 Department of General Surgery, University of Health Sciences-Somalia Turkey Recep Tayyip Erdogan Education and Research Hospital, Mogadishu, Somalia; 3 Department of Orthopaedic Surgery, University of Health Sciences-Somalia Turkey Recep Tayyip Erdogan Education and Research Hospital, Mogadishu, Somalia; 4 Department of Cardiovascular Surgery, University of Health Sciences-Somalia Turkey Recep Tayyip Erdogan Education and Research Hospital, Mogadishu, Somalia

**Keywords:** Terror-related trauma, Blast injury, Improvised explosive device (IED), Gustilo-Anderson, Damage control surgery (DCS)

## Abstract

**Background:**

To describe the severity and types of blast-related extremity injuries and the presence of accompanying vascular injuries (VI) and amputation, and to identify the associated factors affecting the treatment management and clinical course.

**Methods:**

The study included 101 patients with extremity injuries caused by a bomb explosion. The radiographs and computed tomography angiographies of the patients were evaluated in terms of injury patterns, presence of penetrating fragments and fractures, and localization (upper or lower extremity) and type (open or closed) of injury. The Gustilo-Anderson classification was used for open fractures. According to their severity, open fractures classified as types 1 and 2 were included in Group 1 and those classified as type 3A, 3B and 3C in Group 2.

**Results:**

As a result of blast exposure, 101 (57.7%) patients had extremity injuries, of which 76 (75.2%) presented with at least one fracture. Of the total of 103 fractures, nine (8.8%) were closed and 94 (91.2%) were open. Thirty-eight (40.4%) of the open fractures were located in the upper extremities, and 56 (59.6%) in the lower extremities and pelvis. Open fractures were most frequently localized in the femur (*n* = 20; 21.2%), followed by the tibia (*n* = 18; 19.1%). The majority of patients with open fractures were in Group 1 (71.4%). The duration of hospital stay was longer in Group 2 (12.1 ± 5.8 vs. 6.3 ± 6.7 days, *p* <  0.0001, respectively). Mortality among patients in Group 2 (45.0%) was significantly higher than in Group 1 (8.0%) (*p* <  0.0001). Similarly, the injury severity score (ISS) was higher in Group 2 (median 20 vs. 9, *p* <  0.0001). VI was present in 13 (12.9%) of all patients, and amputation in seven (7.9%).

**Conclusion:**

The presence of severe open fractures, VI, and high ISS score can be considered as important factors that increase morbidity and mortality. In extremity traumas, through the secondary blast mechanism, contaminated-fragmented tissue injuries occur. Therefore, we believe that it will be beneficial to apply damage control surgery in places with low socioeconomic level and poor hygienic conditions.

## Background

Terrorist acts and terror-related blasts are frequently experienced in Somalia due to civil war, which has continued for more than a quarter of a century, negatively affecting the entire region. These terrorist events and explosions, which are more frequent in the capital Mogadishu, cause serious injuries, loss of extremities, and deaths. Studies on blast and war traumas reveal that musculoskeletal system injuries constitute 65–70% of all injuries reported from the first world war to the Somalia civil war in 1992 [1, 2]. This is also supported by most of the injuries during the 2003–2014 Iraq and Afghanistan conflicts being observed on the extremities [3, 4].

Although there has been a considerable amount of research on war injuries and military trauma, studies involving injuries in terrorist events targeting civilians are still limited [5, 6]. Two different types of injuries occur as a result of terrorist acts in Somalia as related to gunshot and improvised explosive devices (IEDs).

Blast injuries caused by IEDs occur through multiple mechanisms unlike other traumas. Blast injuries encountered in IED detonations are classified as primary, secondary, tertiary, and quaternary. Primary injuries occur as a result of a high-pressure blast wave while secondary blast injuries develop due to penetrating trauma caused by high-velocity bomb fragments and other debris. Tertiary injuries are blunt traumatic injuries that occur when victims are thrown by the blast wind. Quaternary injuries refer to all remaining injuries due to smoke inhalation and fire [3, 7]. Penetrating injuries due to flying projectiles and bomb fragments are the most common type of injury associated with explosion, with the severity of these injuries ranging from lacerations to traumatic amputations [3]. In order to apply an appropriate treatment, it is important to be aware that the diagnosis and treatment management of musculoskeletal injuries caused by a bomb attack differ from other civilian traumas.

To date, the types and imaging findings of musculoskeletal injuries caused by explosions in many parts of the world have been investigated [5, 6]. However, the anatomical distribution and nature of extremity injuries affecting casualties in Somalia, where explosions are usually intense and require reconstructive and rehabilitative care, have not been studied in detail. This study was performed to describe the severity and types of extremity injuries and the presence of accompanying vascular injuries (VI) and amputations in explosion victims. In addition, it was aimed to determine the associated factors affecting the management and clinical course of these injuries.

## Methods

### Patient data

In this study, the demographic characteristics, medical records, and clinical and radiological data of the patients who were injured during explosions in Mogadishu, the capital of Somalia, between January 2019 and December 2019 were retrospectively analyzed. As a result of the evaluation of 176 patients, 101 with bone fractures of the extremities and only soft tissue injuries and/or VI were included in the study. The remaining 75 patients were excluded from the study due to the absence of extremity injuries on imaging and clinical findings. Local ethics committee approval was received for the study (date: February 12, 2020, number: MSTH-3396).

Laboratory parameters at the time of presentation (hemoglobin and creatinine), number and type of surgical procedures performed, length of hospital stay (LHS), injury severity score (ISS), individual mortality rates, and presence of accompanying non-extremity injuries were recorded. Limb losses proximal to the hand and ankle were included in the amputation group. Amputation was classified as traumatic (on scene), primary (within 24 h), and secondary (surgery after the first intervention).

Multiple fractures of the carpals, metacarpals, phalanges, tarsals and metatarsals were grouped and counted together and evaluated as a single fracture [4]. All patients were also evaluated using ISS, which describes the level of the injury at presentation. This score is calculated using the Abbreviated Injury Severity (AIS) scale, which anatomically divides the body into six regions assigns points from 1 to 6 for each injury and severity. ISS refers to a general score from 0 to 75 indicating the severity of injury. Based on the recommendation of the American College of Surgeons and available literature studies on trauma, we evaluated ISS as follows: minor injury if 1 to 8, moderate if 9 to 15, severe if 16 to 24, and critical if 25 to 75 [8–10].

### Imaging evaluation

The radiographs and computed tomography angiography (CTA) of all included patients at the time of presentation to the emergency department and during their hospital stay were evaluated. In these evaluations, the presence of flying projectiles and bomb fragments, injury patterns, presence of fractures, and the localization (upper or lower extremity) and type (open or closed) of injuries were determined. Soft tissue injury and the presence of VI were also recorded.

According to the Gustilo-Anderson classification (GAC), open fractures were divided into five groups as type 1, type 2, type 3A, type 3B, and type 3C [2]. In addition, in order to determine the relationship between fracture severity and clinical outcomes, the open fractures of GAC types 1 and 2 were included in Group 1, and type 3A, 3B and 3C fractures were included in Group 2 [11, 12].

### Statistical analysis

All analyses were performed using SPSS v. 20.0 (SPSS for Windows 17.0, IL, USA). The variables with normal distribution were shown by mean and standard deviation values. Continuous variables that showed normal distribution were compared using Student’s t-test, whereas those without normal distribution were compared with the Mann-Whitney U test. Categorical variables and frequencies were compared by conducting Pearson’s chi-square (χ2) or Fisher’s exact test. The statistical significance was defined as *p* <  0.05 (two-sided).

## Results

Of the 101 patients included in the study, 76 (75.2%) were male and 25 (24.8%) were female. The mean age of the patients was 32.2 ± 12.7 (median 30; range 1–73) years. In 89 (88.1%) of the patients, injuries occurred through the secondary blast mechanism due to flying projectiles and bomb fragments penetration, while 12 (11.9%) presented with tertiary blast injuries caused by blunt trauma without penetrating fragment were seen.

**Fig. 1:**
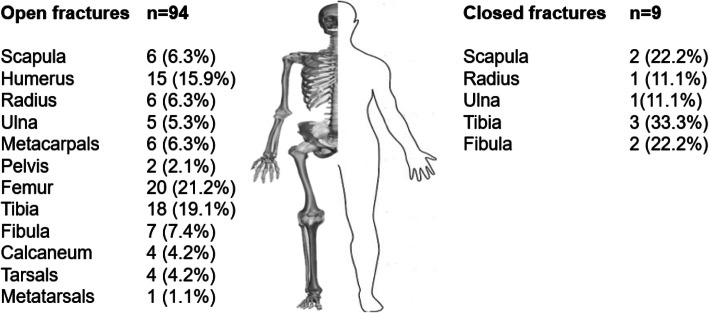
Anatomic distribution of open and closed fractures

At least one extremity fracture was present in 76 (75.2%) patients. Soft tissue injuries without fractures were detected in 25 (24.8%) patients. A total of 103 fractures were detected in 76 patients (some with more than one fracture). There were nine (8.8%) closed fractures in six patients and 94 (91.2%) open fractures in 70 patients. Of the 94 open fractures, 38 (40.4%) were localized the upper extremities and 56 (59.6%) in the lower extremities and pelvis. Open and closed fractures are shown in Fig. 1 according to their localization and number. According to GAC, the most common open fractures were type 2 (51.1%) (Table 1).

**Table 1:** Distribution of the number of open fractures and first treatments according to the Gustilo-Anderson classification

	Conservative	External fixator	Amputation	Total, n (%)
**Type 1**	18	0	0	18 (19.1)
**Type 2**	38	10	0	48 (51.1)
**Type 3A**	3	0	0	3 (3.2)
**Type 3B**	5	10	0	15 (15.9)
**Type 3C**	0	4	7	11 (11.7)
**Total**	64	23	7	94 (100)

As shown in Table 2, the majority of patients with open fractures were in Group 1 (71.4%). The patients in Group 2 had longer hospital stay compared to Group 1 (12.1 ± 5.8 and 6.3 ± 6.7 days, *p* < 0.0001, respectively). Similarly, ISS, number of surgical procedures, mortality rate and VI were found higher in Group 2 (*p* < 0.0001). The hemoglobin value at the time of presentation was significantly lower in Group 2 (*p* = 0.021) (Table 2).

**Table 2:** Demographic and clinical characteristics of patients according to the severity of their open fractures

Variables	Group 1 (Type 1 and 2) (***n*** = 50)	Group 2 (Type 3) (***n*** = 20)	***p*** Value
Gender (male) n (%)	41 (82.0)	13 (65.0)	0.114
Age (years)	33.1 ± 11.6	30.6 ± 14.1	0.532
Vascular injury n (%)	0 (0)	11 (55.0)	< 0.001*
ISS	7.7 ± 5.8	18.6 ± 9.3	< 0.001*
ISS grouping			
Minor (1–8)	13 (26.0)	1 (5.0)	
Moderate (9–15)	29 (58.0)	9 (45.0)	< 0.001*
Severe (16–24)	7 (14.0)	3 (15.0)	
Critical (25–75)	1 (2.0)	7 (35.0)	
Length of hospital stay (days)	6.3 ± 6.7	12.1 ± 5.8	< 0.001*
Hemoglobin (g/dL)	11.4 ± 2.7	8.4 ± 2.9	0.021*
Creatinine (mg/dL)	0.67 ± 0.41	0.68 ± 0.34	0.609
Number of surgical procedures	2.6 ± 0.9	4.5 ± 1.4	< 0.001*
Additional organ injury n (%)	18 (36.0)	6 (30.0)	0.426
Mortality n (%)	4 (8.0)	9 (45.0)	< 0.001*

*ISS* injury severity score

*p < 0.005

Only 13 (12.9%) of 101 patients had VI, and four of these patients underwent CTA. In total, 14 extremities presented with VI. A patient who underwent transhumeral amputation and had a brachial artery injury had rupture in the radial and ulnar arteries in the upper extremity of the opposite side. Accompanying type 3C open fractures, median and radial nerve injuries were also detected in this patient.

**Fig. 2:**
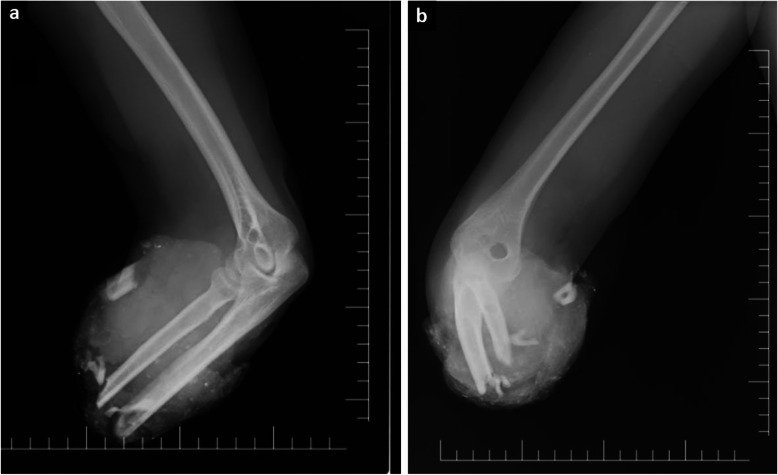
Radiography images of an 18-year-old woman with traumatic amputation. **a-b** Anteroposterior and lateral radiographs showing transradial traumatic amputation, type 3C open fractures in the radius and ulna, extensive soft tissue loss, and fragmented bones

**Fig. 3:**
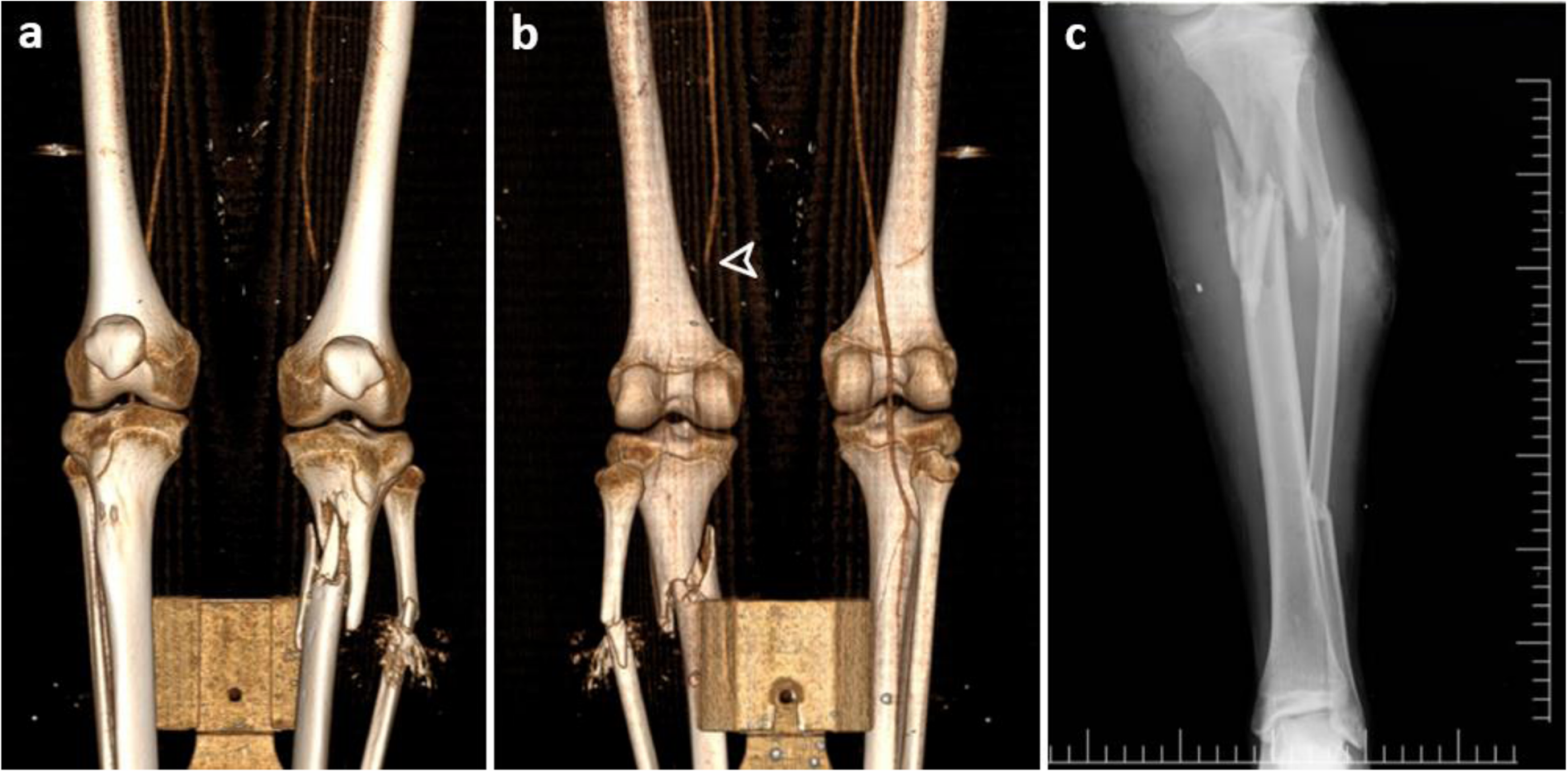
Images of a patient with popliteal artery injury due to open fractures in the tibia and fibula. **a-b** 3D-reconstructed computed tomography angiography images in the anterior and posterior views showing type 3C open fractures in the tibia-fibula and no blood flow in the popliteal artery (arrowhead). **c** Radiography image showing open fractures in the tibia and fibula

**Fig. 4:**
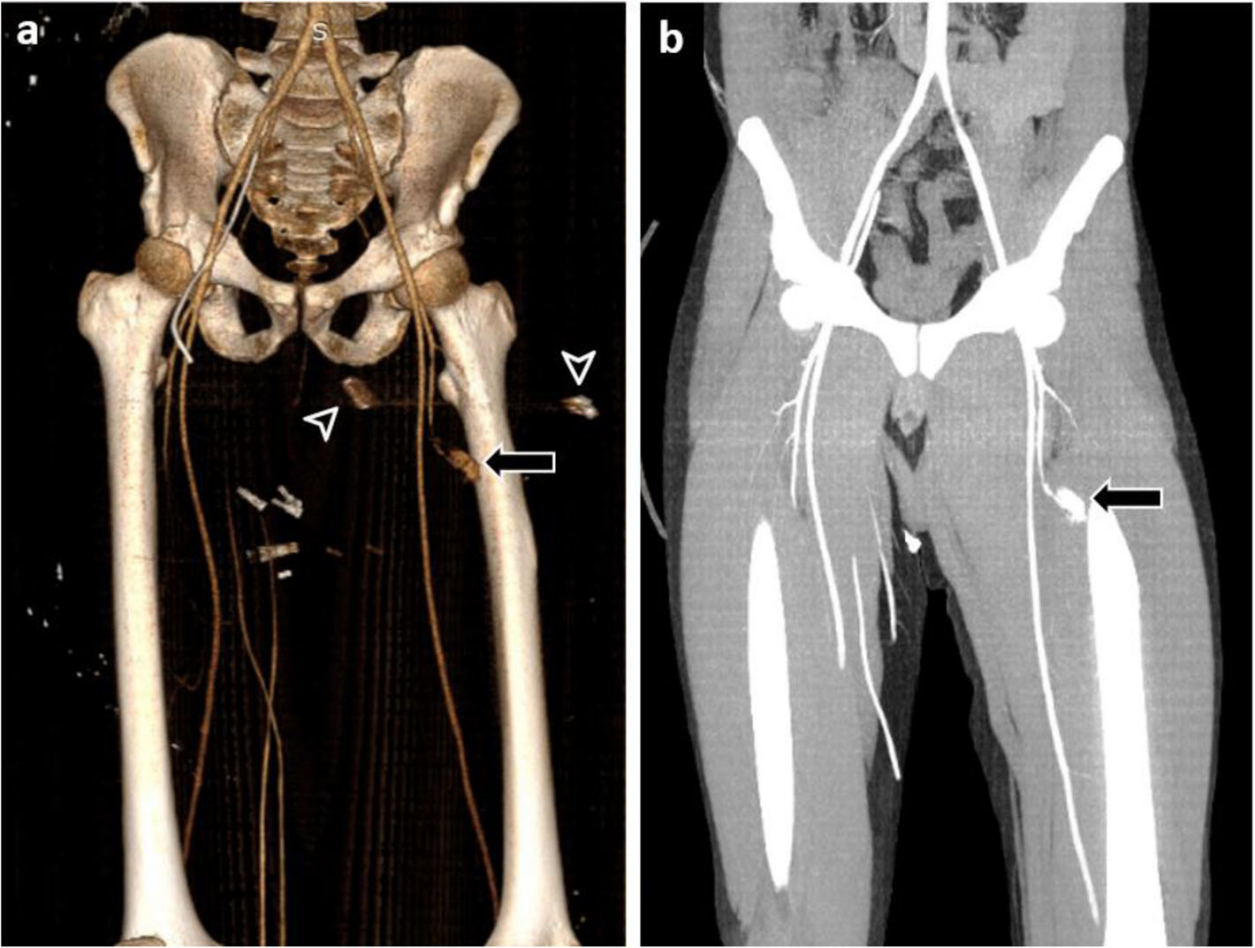
Computed tomography angiography images of a patient with arterial injuries due to bomb fragment. **a-b** Volume-rendered three-dimensional reconstruction and coronal MIP images showing pseudoaneurysm (arrows) and bomb fragment (arrowheads) in the profunda femoris artery. There is no bone fracture in this patient

The localization of amputation was transtibial in three patients, transfemoral in two, and transradial and transhumeral in one case each. There were two patients with traumatic amputation and five that underwent primary amputation (Fig. 2). The clinical and radiological findings and treatments of amputees and non-amputees with VI are shown in Table 3. In addition, the data of two patients with VI are presented in Figs. 3 and 4.

**Table 3:** Clinical and radiological findings of patients with vascular injury

	Arterial injury	Fracture	Treatment	GAC	LHS (days)
**Amputation (*n*** **= 7)**					
Trans-femoral (*n* = 2)	Femoral artery	Femur (*n* = 1)	Traumatic amputation Wound debridement Soft tissue repair	Type 3C	5
	Tibial artery	Tibia (*n* = 1)	Transfemoral amputation was performed due to the wide skin defect in the femur and posterior flap insufficiency.	Type 3C	7
Trans-tibial (*n* = 3)	Tibial artery	Tibia and fibula		Type 3C	5
	Tibial artery	Tibia and fibula		Type 3C	8
	Tibial artery	Tibia and fibula		Type 3C	9
Trans-radial (*n* = 1)	Radial and ulnar arteries	Radius and ulna	Traumatic amputation Wound debridement Soft tissue repair	Type 3C	7
Trans-humeral (*n* = 1)	Brachial artery	Humerus		Type 3C	24
**Non-amputation (*n*** **= 7)**					
Fracture (*n* = 4)	Tibial artery	Tibia and fibula	Saphenous vein graft External fixator	Type 3C	7
	Tibial artery	Tibia and fibula	End-to-end anastomosis External fixator	Type 3C	12
	Popliteal A.	Tibia and fibula	Saphenous vein graft External fixator	Type 3C	18
	Radial and ulnar artery	Humerus and ulna	End-to-end anastomosis External fixator for the humerus External fixator and K wire for the radius and ulna	Type 3C	24
Non-fracture (*n* = 3)					
Femur (*n* = 2)	PFA (A-V fistula) PFA (pseudoaneurysm, extravasation)	None	Surgical fistula repair PFA ligation	–	7 8
Popliteal (*n* = 1)	Popliteal artery	None	Saphenous vein graft	–	32

*GAC* Gustilo-Anderson classification, *PFA* Profunda femoris artery, *LHS* Length of hospital stay

As shown in Table 4, patients with VI had a significantly higher ISS score and longer LHS than those with non-vascular injuries (NVI) (*p* < 0.0001 and *p* = 0.034, respectively). The hemoglobin value (9.8 ± 2.7) of the patients with VI was significantly lower compared to the NVI group (*p* = 0.033). In addition, the creatinine levels were significantly higher in the VI group compared to the NVI group (*p* = 0.042). The patients with VI had a higher mortality rate compared to those with NVI (53.8 vs. 6.9%, respectively, *p* < 0.0001) (Table 4).

**Table 4:** Demographic and clinical characteristics of the patients in vascular injury and non-vascular injury groups

	Vascular injury (***n*** = 13)	Non-vascular injury (***n*** = 88)	***p*** Value
Gender (male)	11 (84.6)	65 (73.9)	NS
Age (years)	32.6 ± 13.3	32.1 ± 12.7	NS
ISS	20.7 ± 9.6	11.1 ± 5.9	< 0.0001
ISS grouping			
Minor (1–8)	0 (0)	38 (43.2)	
Moderate (9–15)	5 (38.5)	39 (44.3)	< 0.001
Severe (16–24)	1 (7.7)	10 (11.4)	
Critical (25–75)	7 (53.8)	1 (1.1)	
LHS (mean of days ± S.D)	11.4 ± 8.2	7.1 ± 6.2	0.034
Hemoglobin (g/dL) at the time of presentation	9.8 ± 2.7	11. 8 ± 2.8	0.033
Creatinine (mg/dL) at the time of presentation	0.99 ± 0.36	0.68 ± 0.32	0.042
Mortality n (%)	7 (53.8)	6 (6.9)	< 0.0001

*LHS* length of hospital stay, *ISS* Injury severity score

**Fig. 5:**
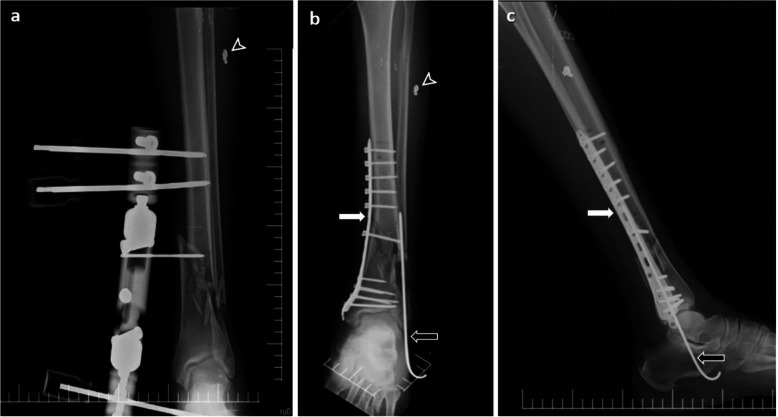
Images of a patient with type 3C open fractures in the tibia and fibula. Radiography image showing **a** the external fixator applied for bone stabilization and **b-c** Plate and screw placed in the tibia (white arrows) and intramedullary wire placed in the fibula (black arrows) for definite treatment. Penetrating fragments (arrowheads) are also present in soft tissue

Seven of the 11 limbs with type 3C fractures were treated by amputation. Bleeding control and primary stump repair were performed in seven patients who underwent amputation. The remaining four type 3C fractures were initially treated by wound debridement and temporary bone stabilization with an external fixator, followed by definitive treatments in the coming weeks (Fig. 5). Treatment methods applied according to fracture types are shown in Table 1.

Fasciotomy was performed in three (3.9%) patients with 76 fractures. Fasciotomy was applied to one patient with popliteal artery injuries and two with tibial artery injuries due to suspected compartment syndrome within the first 24 h.

There were 33 patients (32.7%) presenting with thoraco-abdominal and head and neck injuries in addition to extremity traumas. Seventeen of these patients had thoracic injuries such as pulmonary contusion, pneumothorax, and hemothorax. These were accompanied by liver, spleen and kidney lacerations, and intestinal perforations. Head and neck injuries such as intracranial hemorrhage and maxillofacial bone fractures were detected in 12 patients. In addition, multisystemic injury involving all regions was detected in four patients.

## Discussion

While terrorism was a problem of only underdeveloped and developing countries in the past, it now presents as a global problem affecting innocent civilians worldwide and results in widespread fear, injury, chaos, and death [13]. In recent years, the world has been subjected to the devastating effects of many terrorist attacks, such as the bombings of 2001 New York, 2004 Madrid train, 2005 London metro, 2013 Boston marathon, 2015 Ankara, and 2003–2014 Iraq and Afghanistan conflicts [4, 7, 14, 15].

This study described blast extremity injuries in people exposed to terrorist attacks. The effects of open fracture types and the presence of VI accompanying extremity injuries were shown in terms of the types of surgery performed and LHS. Our data reveal that most of the victims that survived terrorist attacks in recent years present with extremity injuries (Table 5). The data obtained also show that open fractures were significantly higher in blast injuries than closed fractures (91.2% vs. 8.8%). Similarly, in the literature, the rate of open fractures (61.5–83%) is reported to be higher than that of closed fractures [4, 16, 17].

**Table 5:** Incidence of extremity injuries observed in the current study and reported in other sources

Anatomic localization (n, %)	2003–2014 Iraq and Afghanistan conflicts [4]	OIF and OEF 2007 [16]	Current study
Open fracture n (%)	941/1530 (61.5)	758/915, (82)	94/103, (91.2)
*Upper extremities*	344 /941 (36.5)	392/758, (51.7)	38/94, (40.4)
*Lower extremities and pelvis*	597/941 (63.5)	366/758, (48.3)	56/94, (59.6)
Extremity injuries n (%)	1813/2348, (77)	3575/6609, (54)	101/176, (57.7)
Total injuries (n)	2348	6609	176

*OIF* Operation Iraqi Freedom, *OEF* Operation Enduring Freedom

In this study, although 71.4% of open fractures were types 1 and 2 according to GAC, the cases that resulted in amputation and VI were associated with type 3 fractures. In addition, LHS, ISS, and mortality rate were higher in type 3 fractures. This can be explained by blast injuries often occurring through more than one mechanism and affecting multiple systems. The destructive power of bombs depends on the combined effect of the blast wave (primary), bomb fragments and other debris penetration (secondary), and blunt trauma (tertiary) caused by the explosion [13, 15]. Similarly, although the majority of injuries to the extremities consisted of bomb fragments and other debris detected on imaging, the coexistence of extensive tissue loss, severe VI, and 6.9% amputation suggest that blast mechanisms other than secondary may have also been involved. In addition, unpredictable types of injuries may occur due to the effects of nails, screws and metal balls filled in IEDs used in the explosion. Accordingly, multiple penetrating fragments hit different parts of the body, resulting in multiple organ damage and injuries that are difficult to treat. In this study, high ISS and accompanying thoracoabdominal and cranial injuries were found to be factors associated with mortality.

Although open fractures of the extremities can lead to disability, mortality can be significantly reduced without surgery through minimum first aid, fluid supplements, and antibiotic use. With the application of these treatments, blast injuries to the extremities rarely cause death. However, other accompanying systemic injuries, such as thoracoabdominal and cranial injuries have been shown to significantly increase mortality [18]. This is also supported by the presence of accompanying injuries to non-extremity organs and high ISS values in the current study. In such cases, mortality will inevitably develop. In addition, although isolated bone and soft tissue injuries in the extremities are not an important cause of mortality today, they constitute the most important part of surgical load due to their high frequency. Even if mortality does not develop in these patients, they appear as the most common cause of permanent disability in low-income countries, such as Somalia, in which services related to physical rehabilitation and socioeconomic reintegration are not easily accessible at the end of the recovery period.

One of the important factors affecting mortality and healing process in extremity injuries is VI, a condition that requires urgent surgical intervention because it can lead to hemodynamic instability and severe ischemia. In a comprehensive study examining all traumas in Israel between 2000 and 2005, it was reported that 243 (9.85%) of 2466 people injured in terrorist acts had VI, and mortality occurred in 22.2% of these cases [15]. In another study conducted in Israel [5], of the 1261 explosion casualties, 109 were VT victims (8.6%). In the group of critically injured patients (ISS, 25–75), 51.4% had VI, compared with only 15.5% of the NVI patients. Similar to these studies, in our study, 12.9% (*n* = 13/101) of the patients with blast injuries had VI, and critical injury (53.8 vs. 1.1%) and mortality (53.8 vs. 6.9%) were higher in this patient group. Therefore, it can be stated that the presence of VI in extremity injuries directly affects mortality and the clinical course of the patient [13, 15]. In the current study, all patients requiring amputation had VI and accompanying type 3C open fractures. In addition, the patients with VI presented to the hospital with hypovolemic shock and low hemoglobin values compared to NVIs (*p* = 0.033), supporting the idea that VI is a factor associated with mortality. On the other hand, in the presence of multiple fractures, in addition to arterial injuries, bleeding resulting from the bone itself has also been shown to easily lead to a clinical presentation of hypovolemic shock. In addition to any blood loss, burns and tissue destruction are among the factors leading to the development of hypovolemic shock in blast injuries. Large soft-tissue wounds result in considerable tissue edema with further loss of plasma and circulating volume. This causes the worsening of hypovolemic shock and leads to mortality [18].

In a 10-year retrospective review of over 10,000 trauma patients sustaining extremity injury, Branco et al. [19] described a fasciotomy rate of 2.8%. During this period, 315 fasciotomies were undertaken in 237 patients, with 68.4% of the procedures being performed below the knee. According to their results, young males, patients with penetrating or multi-system trauma, those requiring blood transfusion, and those with open fractures or vascular injury (arterial, venous, or combined) were at the highest risk of requiring a fasciotomy after extremity trauma. Similarly, in the current study, fasciotomy was performed in three (3.9%) patients with severe VI due to the development of compartment syndrome in the lower extremities. The rapid increase in the serum creatinine levels of these patients, who were followed up in the intensive care unit, was explained by the occurrence of acute tubular necrosis due to traumatic rhabdomyolysis and hypovolemia. Although hemodialysis was performed on these patients, mortality developed due to multiple organ failure. The creatinine values in the patients with VI in the study cohort being higher than the NVI group even at early stages may be indicative of severe muscle destruction (rhabdomyolysis) and the consequent development of acute renal tubular necrosis. It should not be forgotten that crush injuries and rhabdomyolysis may also occur as a result of blasts, and patients with this hypovolemic condition should receive urgent volume resuscitation [20].

In the treatment of extremities injuries, the most important point to consider is the necessity of performing damage control surgery (DCS) in certain patients. This treatment involves early, marginal and meticulous wound debridement, temporary stabilization of fractures (usually with an external fixator), ensuring physiological recovery, and then performing definitive therapy after the acute phase is completed. Treatment with DCS is primarily aimed at correcting impaired physiology, not anatomy [2, 21, 22]. However, as was the case in this study, the application of DCS treatment in stages is challenging in areas facing frequent terrorist attacks, having insufficient resources, and poor hygienic and socio-economic conditions. Thus, debridement and late primary closure form the basis of surgical treatment. In the treatment of fractures, due to high tissue loss in blast injuries and risk of infection, after the fixation procedure using bone immobilization methods, it is recommended to leave the wound open to provide drainage. In addition, primary closure is not recommended in these patients [2, 13, 21]. It has been suggested that it would be more appropriate to use an external fixator for the first bone immobilization in open fractures (especially types 3B and 3C) and that internal fixation should not be routinely preferred for this type of injury because it can cause a high rate of infection. This was supported by the presence of infection in 50–80% of soldiers in the United States Army during the Vietnam war and the Soviet Army during the Afghanistan war [13].

An important limitation of this study is that the patients who were discharged after the first treatment could not be fully followed up due to their low socioeconomic level and lack of health insurance. In addition, multiple fractures of the carpal and tarsal bones were considered together and grouped as a single fracture, which may be inadequate to differentiate between complex and simple fractures. This can be considered as a limitation, especially in cases requiring amputation, especially that of the lower limb. Despite this, our study presents as a comprehensive work describing blast-related extremity injuries in Somalia.

## Conclusions

Extremity injuries frequently occur as a result of explosions, and their diagnosis and treatment management requires a complex and multidisciplinary approach. The presence of VI and high ISS present as important factors that increase morbidity and mortality, especially in severe open fractures. In extremity traumas, direct bone fractures and indirect contaminated-fragmented tissue injuries occur through the secondary blast mechanism. Therefore, we consider that it will be beneficial to apply DCS methods in areas with a low socio-economic level and poor hygienic conditions.

## Data Availability

All data that are relevant for the study are included in this published article. Further datasets analyzed during the current study are available from the corresponding author on reasonable request. The Figures or Tables used in this study have not been published elsewhere before, and these are the responsibility of the authors. Consent was obtained from the institutional review boards to use the data and to conduct this study.

## Abbreviations

VI: Vascular injury
ISS: Injury severity score
DCS: Damage control surgery
IED: Improvised explosive device
GAC: Gustilo-Anderson classification
LHS: Length of hospital stay
CTA: Computed tomography angiography
PFA: Profunda femoris artery
NVI: Non-vascular injuries
